# The influence of direct transportation to neurosurgical-capable medical centers on the clinical outcomes of patients with spontaneous intracerebral hemorrhage in urban area: a two-center retrospective study

**DOI:** 10.3389/fneur.2025.1659673

**Published:** 2025-08-29

**Authors:** Zijian Chen, Haibo Li, Zhaodi Liao, Xuexiang Shen, Peizhi Qin, Wei Ji, Yuanrun Zhu

**Affiliations:** ^1^Shaoxing TCM Hospital Affiliated to Zhejiang Chinese Medical University, Shaoxing, China; ^2^The Affiliated Wuxi People’s Hospital of Nanjing Medical University, Wuxi, China

**Keywords:** intracerebral hemorrhage, emergency medical service, prehospital transportation, surgical treatment, retrospective study

## Abstract

**Introduction:**

Spontaneous intracranial hemorrhage (ICH) is one of the major causes of morbidity and mortality worldwide due to its poor clinical outcomes. Recent guidelines recommend transferring to medical centers with neurosurgical capabilities to improve outcomes, but it remains unclear whether centers that do not have such neurosurgical capabilities should be bypassed. The current study analyzed the effect of direct transportation to neurosurgical-capable centers on patients with spontaneous ICH in the urban area of Southeast China.

**Methods:**

We included 143 adult patients with spontaneous ICH admitted to two neurosurgical-capable centers from January 2022 to December 2024.

**Results:**

A total of 33 patients were transferred from local centers without neurosurgical capabilities, and 110 of them were admitted directly. The patients had similar baseline characteristics and initial status upon admission. Patients transferred from local centers had a shorter time interval between Emergency Medical Service (EMS) initiation and first computed tomography (CT) scan (0.9 ± 0.3 h vs. 1.7 ± 0.6 h, *p* < 0.001) but a longer time interval before arriving at a neurosurgical-capable center (1.6 ± 0.4 h vs. 1.4 ± 0.6 h, *p* = 0.047). Clinical outcomes, including in-hospital mortality and Glasgow Outcome Scale (GOS) score upon discharge, indicated no statistical difference between the groups, regardless of whether the patients underwent neurosurgical operations or not.

**Discussion:**

In conclusion, the strategy of direct transportation to neurosurgical-capable centers in urban areas did not improve clinical outcomes among patients with ICH; therefore, transfer from local centers after primary diagnosis might be an acceptable strategy.

## 1 Introduction

Intracerebral hemorrhage (ICH) is defined as brain injury attributable to acute blood extravasation into the brain parenchyma from a ruptured cerebral blood vessel (1). Spontaneous ICH is one of the major causes of morbidity and mortality worldwide due to its poor clinical outcome (2–6). In the past, surgical management of ICH lacked reliable evidence, and previous trials of open surgical evacuation indicated negative results (7–10). However, recent studies seemed to change the tide (11). In 2024, the ENRICH study reported that minimally invasive hematoma evacuation could result in improved 180-day outcomes among patients with lobar hemorrhage who underwent neurosurgery within 24 h of an ICH event (12). The SWITCH study also revealed that decompressive craniectomy plus best medical treatment might offer superior outcomes compared to best medical treatment alone among patients with severe deep ICH, although the evidence was relatively weak (13). These novel results highlighted the effectiveness of neurosurgical intervention, especially in the early stage after ICH occurrence, which raised another previously existing question: whether centers that do not have neurosurgical capabilities should be bypassed in the prehospital transportation stage of possible ICH patients. The answer to this question is undetermined (1).

In 2023, one study based on the RACECAT trial reported that transfer protocols bypassing local stroke centers were related to worse functional outcomes among patients with ICH, which provided the first data from a randomized clinical trial about the effect of different prehospital transport protocols (14). The report presented high-level evidence on the topic, but its setting might not be suitable for all situations. In the report, the bypassing strategy is based on endovascular treatment capability, and the local centers are located in non-urban areas that are far away from capable centers. In urban areas, centers with and without neurosurgical capabilities might be less isolated from each other, and for ICH patients, neurological deterioration (ND) in the prehospital stage could be common and dangerous (15–17). It is necessary that further studies in urban areas are performed to provide more evidence on this topic.

In the current study, we intend to analyze the effect of direct transportation to medical centers with neurosurgical capability among patients with spontaneous ICH in the urban area of Southeast China through a two-center retrospective study.

## 2 Methods

### 2.1 Study design and patient population

This retrospective study included adult patients (aged 18 years and older) with spontaneous ICH admitted to two medical centers from January 2022 to December 2024. The two medical centers are neurosurgical-capable centers and are both located in urban areas in Southeast China (Wuxi City and Shaoxing City). The emergency neurosurgical interventions included external ventricular drain (EVD) or intracranial pressure (ICP) monitor placement, craniectomy/craniotomy, hematoma evacuation, and other essential procedures (such as minimally invasive neuroendoscopy). The sample size was based on the available data. For patients directly transported to neurosurgical-capable centers, symptoms should be acute (symptom onset and noticed within 1 h), and the patients should be brought to the hospital by Emergency Medical Service (EMS) vehicles. Stable patients who were admitted for rehabilitation treatment or patients who arrived at the hospital by non-emergency means were not included. For patients transferred from lower-level centers, the transfer decision should be made immediately after ICH diagnosis is confirmed (by a positive neuroimaging result), and the transfer should also be carried out by the EMS vehicle. Patients transferred through a non-emergency approach were not included either. The patients were divided into two groups based on their prehospital transfer procedure: direct transportation to centers with neurosurgical capability or transfer from local centers after initial diagnosis.

The exclusion criteria included (1) discharged or died within 48 h; (2) confirmed cerebrovascular diseases caused ICH by computed tomography angiography (CTA), magnetic resonance angiography (MRA), or digital subtraction angiography (DSA) examination (for example, aneurysm or arterio-venous malformation (AVM)); (3) unavailable medical history or lack of essential EMS transfer information; and (4) repeated ICH events within 3 months. The initial treatment, decision to transfer, and the following neurosurgical intervention (if applicable) were performed following current international and regional guidelines (1, 18).

### 2.2 Transfer details

**Figure 1:**
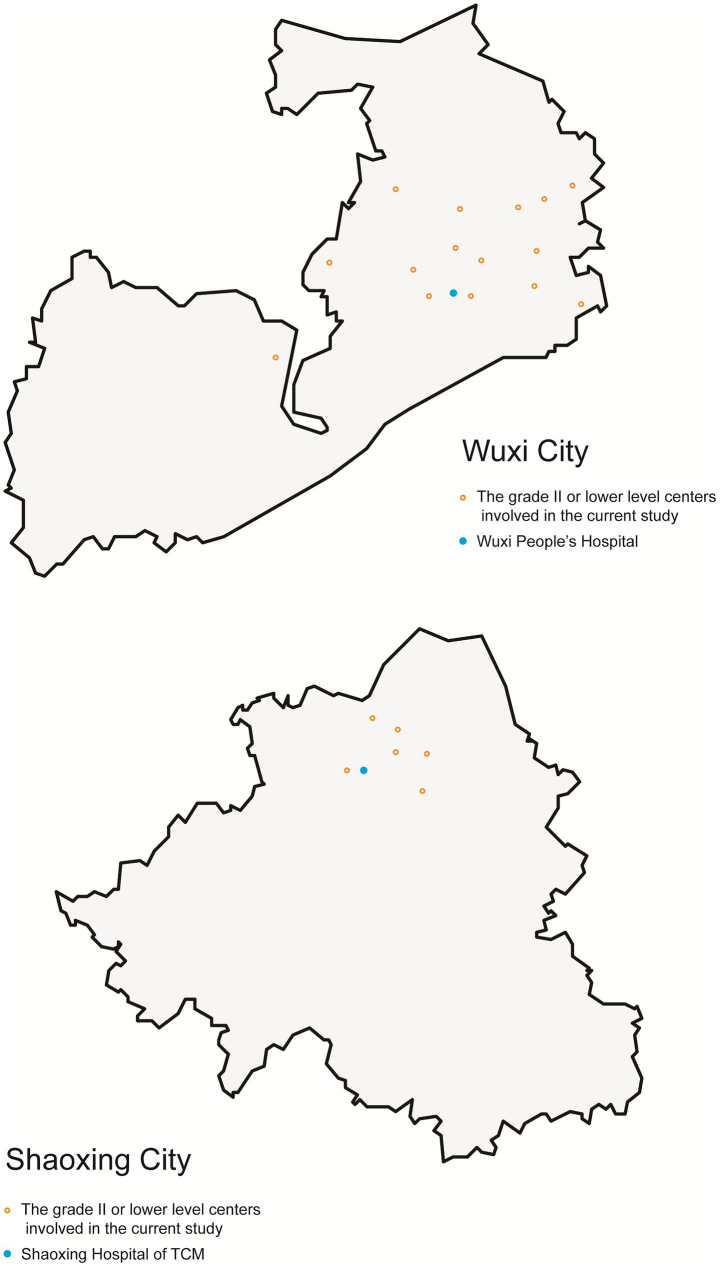
The distribution of the medical centers involved in the current study. This figure presents the relative position of the two medical centers with neurosurgical capabilities and other lower-level centers. The figure does not reflect the accurate distance between these centers or the geological area of the cities.

The two medical centers with neurosurgical capacity in the study were both located in urban areas of the cities. Patients transferred to these two medical centers were from 21 different lower-level centers. The distribution of the lower-level centers is presented in Figure 1. All patients included in the current study were transferred using an EMS vehicle, and no air transfer was involved. The EMS vehicle involved was not equipped with mobile CT or any other radiological equipment in both cities.

### 2.3 Data collection

We retrospectively obtained clinical data from the medical records, which included sex, age, GCS upon admission, transfer time and distance, time interval from EMS initiation to arrival at first medical facility (local or neurosurgical capable center), time interval from EMS initiation to first neuroimaging scan, medical history, vital signs upon admission, pupils, hematoma volume and its dynamic change, ventilation duration, ICU stay, and operation details (if applicable). Clinical outcomes, including in-hospital mortality and Glasgow Outcome Scale (GOS) upon discharge, were documented (1 - death, 2 - persistent vegetative state, 3 - severe disability, 4 - moderate disability, and 5 - good recovery). The GOS scores were evaluated by senior neurosurgeons according to existing reviews (19). Hematoma expansion was defined as an increase of 33% or 6 mL or more in hematoma volume according to repeat CT/MRI images within 24 h after the initial scan (14).

No personal information is traceable in the current report. The study is in keeping with the ethical standards of the 1964 Declaration of Helsinki and its later amendments. The study was approved by the ethics committee of both medical centers involved in the study (project number KY25033).

The two participating sites adhere to the same standardized data collection, processing, and quality control protocol to ensure uniformity. The results were verified by investigators from both centers before submission to guarantee consistency.

### 2.4 Statistical analysis

Statistical analysis was performed using SPSS software version 19.0 (IBM Corp., Armonk, NY, US). Baseline characteristics and clinical outcomes were compared between the direct transport and transfer groups. Normally distributed continuous variables were given as means and standard deviations, and independent samples t-test was performed for comparison. Medians with upper and lower quartiles were given if continuous variables were not normally distributed, and the Mann–Whitney U-test was performed for comparison. Categorical variables were given as numbers and percentages, and the chi-squared (*χ*^2^) test was performed for comparison. We used a cumulative ordinal logistic regression model to evaluate the GOS upon discharge. The adjusted odds ratio (OR) and 95% CI were calculated and reported. To evaluate in-hospital mortality, we performed a Cox proportional hazards regression analysis. Adjusted hazard ratio (HR) and 95% CI were reported. Confounders controlled in the regression models were sex, age, GCS, time for first CT scan, blood pressure, lung infection, pupil abnormity, and hematoma volume. The Hosmer–Lemeshow test was used to verify the regression models. Statistical significance was considered at a *p*-value of < 0.05.

## 3 Results

### 3.1 Patient inclusion and basic characteristics

**Figure 2:**
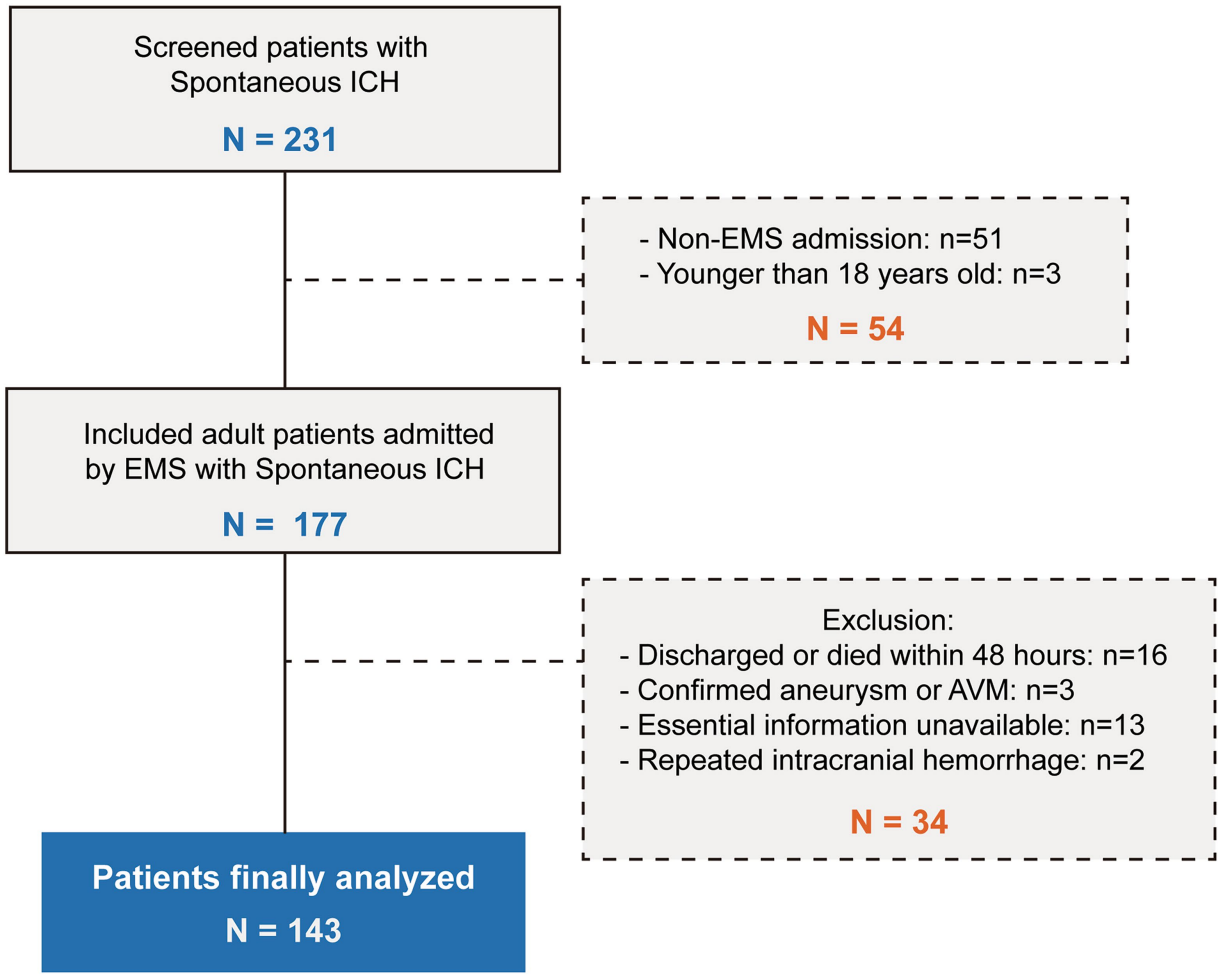
The flowchart of the inclusion and exclusion details. A total of 231 patients were screened and 143 patients were finally included.

We screened a total of 231 spontaneous ICH patients. A total of 51 patients were not admitted through EMS, and 3 patients were younger than 18 years old. A total of 34 patients met the exclusion criteria, and 143 ICH patients were analyzed. The inclusion and exclusion details are summarized in Figure 2.

Among the 143 patients analyzed, 33 were transferred from local low-level centers without neurosurgical capabilities, and 110 were admitted directly. The patients had similar baseline characteristics, including sex, age, and known medical history of hypertension. Patients transferred from local centers had a shorter time interval between EMS initiation and first CT scan (0.9 ± 0.3 h vs. 1.7 ± 0.6 h, *p* < 0.001). The patients also had similar status of admission, including GCS upon admission, blood pressure (both systolic and diastolic), pneumonia, pupil abnormality, original hematoma volume, and hematoma expansion. The ratio of patients who underwent neurosurgical operations was similar (specified types of operations are presented in Table A1), as well as the duration of ventilation, ICU stay, and hospital stay. The characteristics are summarized in Table 1.

**Table 1:** Clinical characteristics between patients transferred from local centers and directly transported to neurosurgical-capable centers.

Patient characteristics	Patients transferred from local centers (*n* = 33)	Patients directly transported (*n* = 110)	*p*
Sex, *n* (%)			0.265
Male	21 (63.6)	81 (73.6)	
Female	12 (36.4)	29 (26.4)	
Age (year), mean ± SD	55.1 ± 13.4	56.6 ± 14.1	0.595
GCS upon admission, mean ± SD	10.2 ± 4.0	9.2 ± 4.3	0.217
^*^Time of first CT scan (hour), mean ± SD	0.9 ± 0.3	1.7 ± 0.6	**<0.001**
Known history of hypertension, n (%)	20 (60.6)	82 (74.5)	0.120
^#^Systolic BP (mmHg), mean ± SD	166.5 ± 30.4	168.0 ± 28.6	0.785
^#^Diastolic BP (mmHg), mean ± SD	94.3 ± 14.8	96.0 ± 22.7	0.679
Pneumonia upon admission, n (%)	9 (27.3%)	24 (21.8%)	0.591
Pupil abnormality, n (%)	5 (15.2%)	27 (24.5%)	0.256
Hematoma volume (mL), mean ± SD	37.9 ± 20.5	37.2 ± 25.2	0.891
Neurosurgical operation, n (%)	24 (72.7%)	70 (63.6%)	0.335
Hematoma expansion, n (%)	2 (6.1%)	8 (7.3%)	0.811
Duration of ventilation (day), median (IQR)	1 (0, 7)	2 (0, 6)	0.141
Duration of ICU stay (day), median (IQR)	6 (0, 17)	5 (0, 16)	0.360
Duration of hospital stay (day), median (IQR)	24 (18, 30)	23 (15, 30)	0.450

SD, Standard deviation; GCS, Glasgow coma scale; CT, Computer tomography; BP, Blood pressure; IQR, Interquartile range; ICU, Intensive care unit. *From EMS initiation. #Upon admission to neurosurgical-capable center. The *p* values are bold when they are < 0.05.

### 3.2 Transfer details

The transfer time of the patients between centers was 0.6 ± 0.3 h, and the transfer distance was 19.0 ± 12.1 kilometers. The transfer distance was recorded as actual traffic distance instead of geological straight-line distance. The overall time between EMS initiation and admission to a neurosurgical-capable center was shorter among patients transported directly (1.4 ± 0.6 h vs. 1.6 ± 0.4 h, *p* = 0.047). The transfer details are presented in Table 2.

**Table 2:** Traffic time and distance of the patients’ transfer between centers.

Transfer details	Patients transferred from local centers (*n* = 33)	Patients bypassing local centers (*n* = 110)	*p*-value
Transfer time between centers (hour), mean ± SD	0.6 ± 0.3	NA	
Transfer distance between centers (km), mean ± SD	19.0 ± 12.1	NA	
Overall time before admission (hour), mean ± SD	1.6 ± 0.4	1.4 ± 0.6	**0.047**
Overall time before admission ≥ 1.6 h, n (%)	18 (54.5%)	36 (32.7%)	**0.023**

SD, Standard deviation; NA, Not applicable. The *p* values are bold when they are < 0.05.

### 3.3 Clinical outcomes

**Figure 3:**
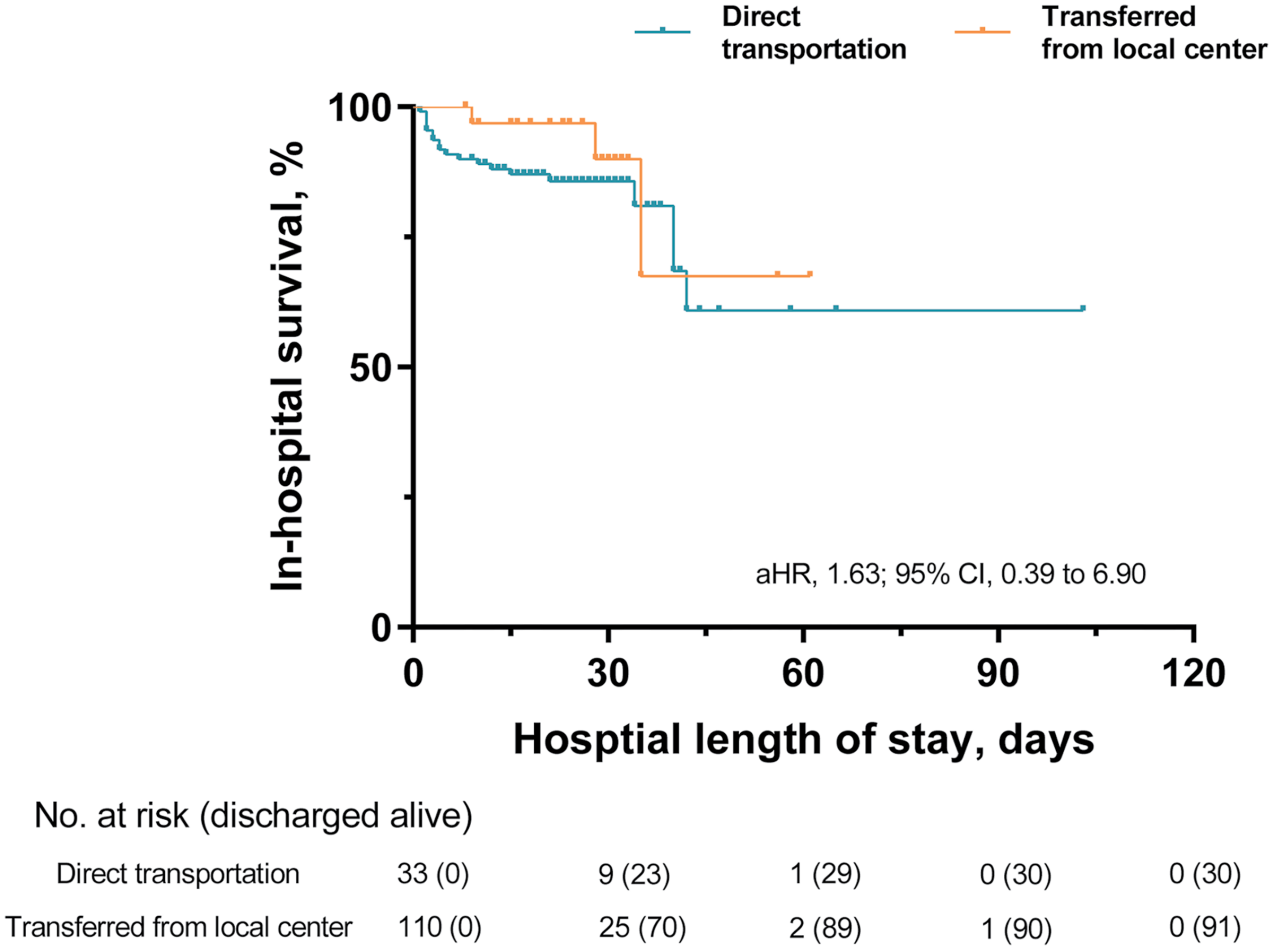
Kaplan-Meier survival curves. In-hospital mortality was evaluated using a Cox proportional hazards regression model. aHR: adjusted hazard ratio.

The clinical consequences of patients with ICH are summarized in Table 3. The in-hospital mortality indicated no statistical difference between the two groups after adjusting for the Cox regression model (17.3% vs. 9.1%, adjusted HR: 1.63, 95% CI: 0.39 to 6.90). Mortality curves (presented in Figure 3) indicated no significant divergence between the two groups, either. Most of the patients were discharged from the hospital or died within 30 days after admission.

**Table 3:** Comparison of clinical outcomes between patients transferred from local centers and those directly transported.

Clinical outcomes	Patients transferred from local centers (*n* = 33)	Patients directly transported (*n* = 110)	Effect variable	Unadjusted value (95% CI)	^*^Adjusted value (95% CI)	^*^p
In-hospital mortality, *n* (%)	3 (9.1%)	19 (17.3%)	HR	1.85 (0.55 to 6.28)	1.63 (0.39 to 6.90)	0.504
GOS upon discharge, mean (SD)	2.94 (1.14)	3.03 (1.27)	Common OR	1.21 (0.60 to 2.42)	1.69 (0.70 to 4.12)	0.245

CI, Confidence interval; OR, Odds ratio; HR, Hazard ratio; GOS, Glasgow outcome scale; SD, standard deviation. *Adjusted odds ratio and hazard ratio control for: sex, age, GCS, time for first CT scan; blood pressure, lung infection, pupil abnormity, and hematoma volume.

**Figure 4:**
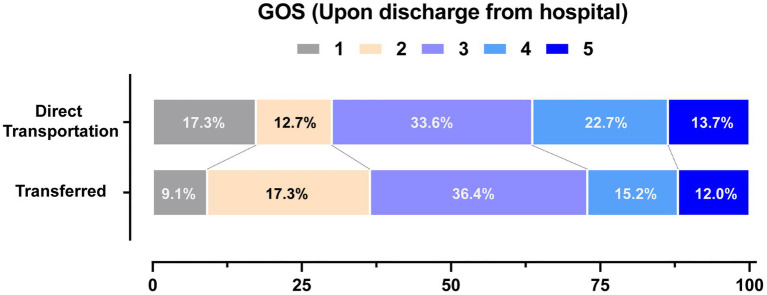
Distribution of the outcomes upon discharge. Distribution of Glasgow outcome scale (GOS) scores upon discharge are presented. The GOS scores range from1 to 5 (1 Dead; 2 Vegetative state; 3 Severe disability; 4 Moderate disability; 5 Good recovery).

The patients of the two groups also had similar GOS scores upon discharge (3.03 vs. 2.94), and no statistical difference was found after being adjusted by a cumulative ordinal logistic regression model (adjusted OR: 1.69, 95% CI: 0.70 to 4.12). The GOS details upon discharge are presented in Figure 4. The patients of two groups had a similar ratio of relatively good outcomes (GOS 4–5, 36.4% vs. 27.2%).

To evaluate the patients who underwent neurosurgical operation, we performed a stratified analysis of clinical outcomes. The results were similar. For patients who underwent neurosurgical operation, the in-hospital mortality (adjusted HR: 1.41, 95% CI: 0.42 to 4.70) and GOS (adjusted OR: 2.34, 95% CI: 0.46 to 11.80) presented no statistical difference between the patients directly transported to neurosurgical-capable centers or those transferred from local centers. For patients who did not need an operation, the in-hospital mortality was not-applicable for analysis since no patients in this layer died. The GOS upon discharge (adjusted OR: 3.37, 95% CI: 0.43 to 26.31) indicated no statistical difference either. The results of stratified analyses are presented in Table 4.

**Table 4:** Stratified analysis of clinical outcomes (stratified by neurosurgical operation).

Layer	Clinical outcomes	Patients transferred from local centers	Patients directly transported	Effect variable	^*^Adjusted value (95% CI)	^*^p
Neurosurgical operation, *N* = 94	In-hospital mortality, n (%)	3 (12.5%)	19 (27.1%)	HR	2.34 (0.46 to 11.80)	0.303
	GOS upon discharge, mean (SD)	2.37 (1.02)	2.54 (0.98)	Common OR	1.41 (0.42 to 4.70)	0.576
No operation needed, *N* = 49	In-hospital mortality, *n* (%)	NA	NA	HR	NA	NA
	GOS upon discharge, mean (SD)	4.00 (0.87)	4.18 (0.71)	Common OR	3.37 (0.43 to 26.31)	0.246

CI, Confidence interval; OR, Odds ratio; HR, Hazard ratio; GOS, Glasgow outcome scale; SD, Standard deviation. *Adjusted odds ratio and hazard ratio control for: sex, age, GCS, time for first CT scan; blood pressure, lung infection, pupil abnormity, and hematoma volume.

## 4 Discussion

In this small-scale retrospective study, direct transportation to a neurosurgical-capable center compared with transfer from local centers resulted in similar in-hospital mortality and GOS scores upon discharge. Direct transportation led to a delayed neuroimaging scan but shortened the time interval between EMS initiation and final admission to neurosurgical-capable centers. The overall in-hospital mortality was 15.4% (after exclusion of patients who died within 48 h), which was similar to previous reports (20).

The 2022 American Heart Association and American Stroke Association (AHA) guidelines for the management of patients with spontaneous ICH indicated that further study was needed regarding whether the destination of patients with potential ICH should be the same as that for patients with large vessel occlusion strokes, or whether centers that do not have neurosurgical capabilities should be bypassed (1). In 2023, a secondary analysis of the RACECAT trial indicated that direct transportation to an EVT-capable center would cause worse clinical outcomes (14). The current study did not indicate any differences in mortality and short-term functional outcomes. One of the major differences between the two studies was the area in which the study was performed. Compared with the current study, the RACECAT trial was carried out in a non-urban area, in which EVT-capable centers were far from local centers. The patients in the secondary analysis of the RACECAT trial had a longer time from symptom onset to hospital arrival (135 min to EVT-capable center and 94 min to local center compared with 84 min to neurosurgical-capable center in the current study). The longer transfer time might be related to a higher risk of transfer complications. It was reported that patients directly transported to an EVT-capable stroke center had a higher probability of experiencing complications during transportation, such as vomiting, and also had a higher probability of in-hospital pneumonia (14). The complications and pneumonia were considered to partly cause the worse clinical outcomes. In other studies, vomiting was reported to be a risk factor for aspiration pneumonia (21). ND events in pre-hospital scenarios were also reported to be common and related to poor outcomes in ICH patients (16, 17). In the current study, the risk of developing pneumonia was similar between the two groups of patients, which might be explained by the relatively shorter transfer time. Unfortunately, due to the retrospective nature, we could not obtain records about transfer complications for all patients in the current study, which made it impossible to analyze the risk of complications.

Surgical treatment of ICH is a topic receiving continuous interest. Recent studies have provided positive evidence regarding the neurosurgical treatment of ICH (11–13). The 2022 AHA guidelines recommended that patients with spontaneous ICH and clinical hydrocephalus should be transferred to centers with neurosurgical capabilities for definitive hydrocephalus management (1, 22, 23). We found that the percentage of patients treated surgically was similar (63.6% vs. 72.7%). Although the directly transported patients had a shorter time from EMS initiation to arrival at a neurosurgical-capable hospital, the clinical outcomes for patients who actually underwent operation were similar between the two groups. These results indicated that, even for patients with ICH who needed surgical treatment, transfer to a neurosurgical-capable center after primary diagnosis in the local center is an acceptable strategy. This approach might be meaningful in preventing high-level medical centers from being overwhelmed, especially in China, which has relatively limited healthcare resources compared with other developed countries (24, 25).

The initial blood pressure-lowering treatment is of great value in ICH. Initiating treatment within 2 h of ICH onset and reaching the target within 1 h can improve functional outcomes (1, 26, 27). In the current study, patients transferred from local centers and directly transported to surgical-capable centers had similar blood pressure levels when arriving at the neurosurgical-capable center. Theoretically, patients transferred from local centers would be diagnosed earlier (they had a shorter time interval before getting the first neuroimaging scan) and should receive blood pressure-lowering treatment before they arrived at a high-level center. The results did not support such a hypothesis. This finding might be related to limited resources during transportation and short dwelling time in local centers. Apart from the earlier initiation of blood pressure control, the shorter time interval before a neuroimaging scan could lead to other potential benefits. For instance, earlier interventions to reverse the effect of anticoagulant medications, as well as respiratory and hemodynamic stabilization, were optimal in the initial management of ICH patients (1). Differential diagnosis is also dependent on neuroimaging results. The management of other diseases with neurological symptoms (for example, ischemic stroke or bacterial meningitis) could be time-sensitive as well (28, 29). Considering the values of earlier neuroimaging scans, creating coordinated monitoring protocols and improving the immediate care and management of these patients at local facilities would be helpful.

The current study has a few limitations. First, as a retrospective study, some factors could not be analyzed due to a lack of data, such as transfer complications and ND events during transportation. These factors might also influence the clinical outcomes. Second, the patient population was limited, which could lower the power of statistical analysis, especially in the stratified analysis. The wide confidence intervals for in-hospital mortality and GOS outcomes suggested insufficient power. These findings should be interpreted with caution. Third, the study focused on the urban area of south-east China. The findings should not be applied directly outside the area. Fourth, long-term follow-up was not a part of the study protocol. Neurological functional outcomes after 3 or 6 months were important for ICH patients but were not analyzed in the current study.

## 5 Conclusion

Patients with ICH directly transported to neurosurgical-capable centers had a prolonged time interval from EMS initiation to the first neuroimaging scan. The interval between EMS initiation and admission to neurosurgical-capable centers was shortened. With an average transfer time of 0.6 h, the strategy of direct transportation to neurosurgical-capable centers in the urban area did not improve clinical outcomes among patients with ICH. Transfer from local facilities after primary diagnosis might be an acceptable strategy.

## Data Availability

The raw data supporting the conclusions of this article will be made available by the authors, without undue reservation.

## Appendix

**Table A1:** Specified neurosurgical interventions of ICH patients.

Neurosurgical interventions, *n* (%)	Patients transferred from local centers (*N* = 33)	Patients directly transported (*N* = 110)
No intervention	40 (36.4%)	9 (27.3%)
Craniotomy/craniectomy and hematoma evacuation	19 (57.6%)	58 (52.7%)
Minimally invasive neuroendoscopy	1 (3.0%)	4 (3.6%)
EVD and/or ICP monitor placement only	4 (12.1%)	8 (7.3%)
*p* value*	0.273	

ICH, Intracranial hemorrhage; EVD, External ventricular drain; ICP, Intracranial pressure. *The *p* value here was calculated using Linear-by-lineal test since 3 data sets were lower than 5.
